# Flow fluorometry quantification of anion channel VRAC subunit LRRC8A at the membrane of living U937 cells

**DOI:** 10.1080/19336950.2020.1730535

**Published:** 2020-02-20

**Authors:** Valentina Yurinskaya, Nikolay Aksenov, Alexey Moshkov, Tatyana Goryachaya, Ashley Shemery, Alexey Vereninov

**Affiliations:** a Institute of Cytology, Russian Academy of Sciences, St-Petersburg, Russia; b School of Biomedical Sciences, Kent State University, Kent, OH, USA

**Keywords:** Anion channel, VRAC, LRRC8A, surface expression, flow cytometry, antibody

## Abstract

Assessing the expression of channels on the cell membrane is a necessary step in studying the functioning of ion channels in living cells. We explore, first, if endogenous VRAC can be assayed using flow cytometry and a commercially available antibody against an extracellular loop of the LRRC8A, also known as SWELL1, subunit of the VRAC channel. The second goal is to determine if an increase in the number of VRAC channels at the cell membrane is responsible for an increase in chloride permeability of the membrane in two well-known cases: during staurosporine (STS)-induced apoptosis and after water balance disturbance caused by hypotonic medium. Human suspension lymphoid cells U937 were used as they are suitable for flow fluorometry and because we have recently studied their membrane chloride permeability during apoptosis. We found that surface expression of endogenous LRRC8A subunits can be quantified in living U937 cells using flow fluorometry with the Alomone Lab antibody. Further, we revealed that treatment of cells for 1 hour using STS or a hypotonic solution did not change the number of LRRC8A subunits to the extent that would correspond to changes in the membrane chloride permeability determined by ion content analysis. This indicates that prolonged increase in chloride permeability of the cell membrane during apoptotic cell shrinkage or cell volume regulation under hypotonicity in U937 cells occurs without altering cell surface expression of VRAC.

## Introduction

Chloride channels are key players in regulating ionic and water balance in animal cells, as chloride is the main external anion for most cells and chloride channels are the main electroconductive pathway for this ion through the cell membrane [1–3]. Volume regulated anion channel (VRAC) is a ubiquitously expressed chloride channel that has attracted much attention since the molecular structure of VRAC has been identified [4–6]. A growing body of evidence indicate that VRAC and their obligatory subunit, LRRC8A have critical roles in many cell functions including cell motility, proliferation, apoptosis, drug and metabolite transport, angiogenesis, and spermatid development, as well as in cell pathophysiological cell functions such as cancer drug resistance, ischemic brain edema, and glaucoma [7–17]. While the molecular structure of VRAC is well documented [18], understanding how VRAC expression at the membrane is regulated has been poorly explored due to lack of appropriate methodology. Electrophysiological methods are suitable for investigating biophysical properties of channels in a single cell and during short-time events but are unsuitable for studying cell populations and the long-term alteration of cells. Methods for quantifying VRAC channels at the cell membrane are critical. The LRRC8A subunit is an indispensable component of VRAC and its number corresponds to the number of whole complexes. The Alomone Lab generated a novel, commercially available antibody against an external epitope of the LRRC8A subunit. Though promising, the use of this antibody to quantify cell membrane VRAC expression on living cells has not been explored until now.

The human lymphoma U937 cell line is widely used to investigate fundamental cell processes like apoptosis, proliferation, cell volume regulation, and anticancer drug resistance. Recently, we identified changes in major pathways of monovalent ion transfer across the plasma membrane of U937 cells during apoptosis caused by staurosporine (STS). Specifically, a 5-fold increase in chloride channel permeability was found at the early stage of apoptosis along with a decrease in Na/K pump activity and changes in potassium and sodium channel permeability [3]. These findings are consistent with many electrophysiological studies which also show an increase in chloride current at the early stage of apoptosis, as well as during hypotonic stress, accompanied by the regulatory volume decrease (RVD) response [19]. However, it is unknown if cell membrane expression of VRAC is altered to facilitate these cellular responses due to lack of appropriate methodology. Therefore, we validated the use of flow cytometry with a novel VRAC LRRC8A subunit antibody to estimate cell-surface VRAC abundance. Further, because VRAC regulates chloride flux and cell volume responses, we investigated whether STS-induced apoptosis or hypotonic stress in U937 cells alter the expression of cell-surface VRAC using our validated flow cytometry method.

Our results show that endogenous VRAC can be quantified in living cells with average native expression levels by flow fluorometry using the Alomone Lab antibody for the LRRC8A subunit. Cell fluorometry using microscope did not appear to be sensitive enough for quantification of the LRRC8A subunit under the same Ab concentration. Quantification of the LRRC8A subunits via flow fluorometry revealed that membrane-bound VRAC expression did not change following STS-induced apoptosis or hypotonicity to the extent that would correspond to changes in the membrane chloride permeability determined by ion content analysis [3].

## Materials and methods

### Cell cultures and media

The human histiocytic lymphoma cell line U937 was obtained from the Russian Cell Culture Collection (Institute of Cytology, Russian Academy of Sciences, cat. number 220B1). Cells were cultured in RPMI 1640 medium (Biolot, Russia) supplemented with 10% fetal bovine serum (HyClone Standard, USA) at 37°C and 5% CO_2_. Cells were grown to a density of 1 × 10^6^ cells per ml. Apoptosis was induced by incubating cells in 1 µM staurosporine (STS, Sigma-Aldrich, Germany) for 1 h. The hypotonicity-induced cell response was studied by incubating cells for 1 h in a hypotonic medium (200 mosM) prepared by mixing RPMI without NaCl with standard RPMI medium. The osmolarity of solutions was checked with the Micro-osmometer Model 3320 (Advanced Instruments, USA).

### Antibodies

Polyclonal Anti-LRRC8A rabbit antibody from Alomone Lab (Jerusalem, Israel, Cat #: AAC-001) was used to visualize volume regulated anion channels (VRAC). These antibodies (LRRC8A-Ab) were raised against an external epitope of LRRC8A, corresponding to the peptide sequence (C)DTGPTGIKYDLDRH and amino acid residues 91–104 of the first extracellular loop of human LRRC8A (Accession Q8IWT6). The cells were incubated with LRRC8A-Ab antibody at room temperature for 30 min by adding 3 µl (2.4 µg) into 50 µl of cell suspensions in living media followed by incubation with the secondary goat anti-rabbit Alexa Fluor 568 antibody (Invitrogen, A21069; 1.5 µl (3 µg) into 50 µl of cell suspensions) in RPMI with 2% BSA for 10 min at room temperature in the dark. After each antibody incubation, cells were washed in PBS + 2% BSA then the cells were resuspended in 200 µl RPMI.

### Fluorescent probes

Fluorescent probes di-4-ANEPPS and CFSE (Sigma-Aldich) were dissolved in DMSO at 1 and 5 mM concentrations, respectively. For cell staining, fluorescent probes were added after intermediate dilution into 0.3 ml of cell suspension, with cell concentration about 1 × 10^6^ cells per ml, at a final concentration of 1 µM ANEPPS or 5 µM CFSE. Samples were incubated for 20–30 minutes at 37ºC.

### Flow cytometry

Fluorescence was analyzed on a CytoFLEX Flow Cytometer (Beckman Coulter Inc., CA, USA). Ab stained cells were excited by 561 nm laser and their fluorescence was captured using a 610/20 nm bandpass emission filter (ECD channel). Cells stained with the other fluorescent probes were analyzed using excitation at 488 nm and different bandpass emission filters: 690/50 nm for ANEPPS (PC5.5 channel) and 525/40 nm for CFSE (FITC channel). All compared histograms were obtained at the same cytometer settings. The area (A) of flow cytometer parameters was used (e.g. FSC-A, SSC-A, ECD-A). We are most interested in the main subsets of P1 and P0, and we prefer to use contour plots taking into account the rule: “Dot plots can be more advantageous when viewing rare populations. Contour plots are more effective with high event counts, because the relative frequency of events is displayed in more detail”. Since many readers prefer dot plots, some basic data is also given in this form.

### Statistical analysis

Data analysis and statistics was performed using CytExpert 2.0 Beckman Coulter software. At least 20,000 events were acquired using the flow cytometer.

## Results

### Cell particle distribution of original U937 cell culture

**Figure 1.**
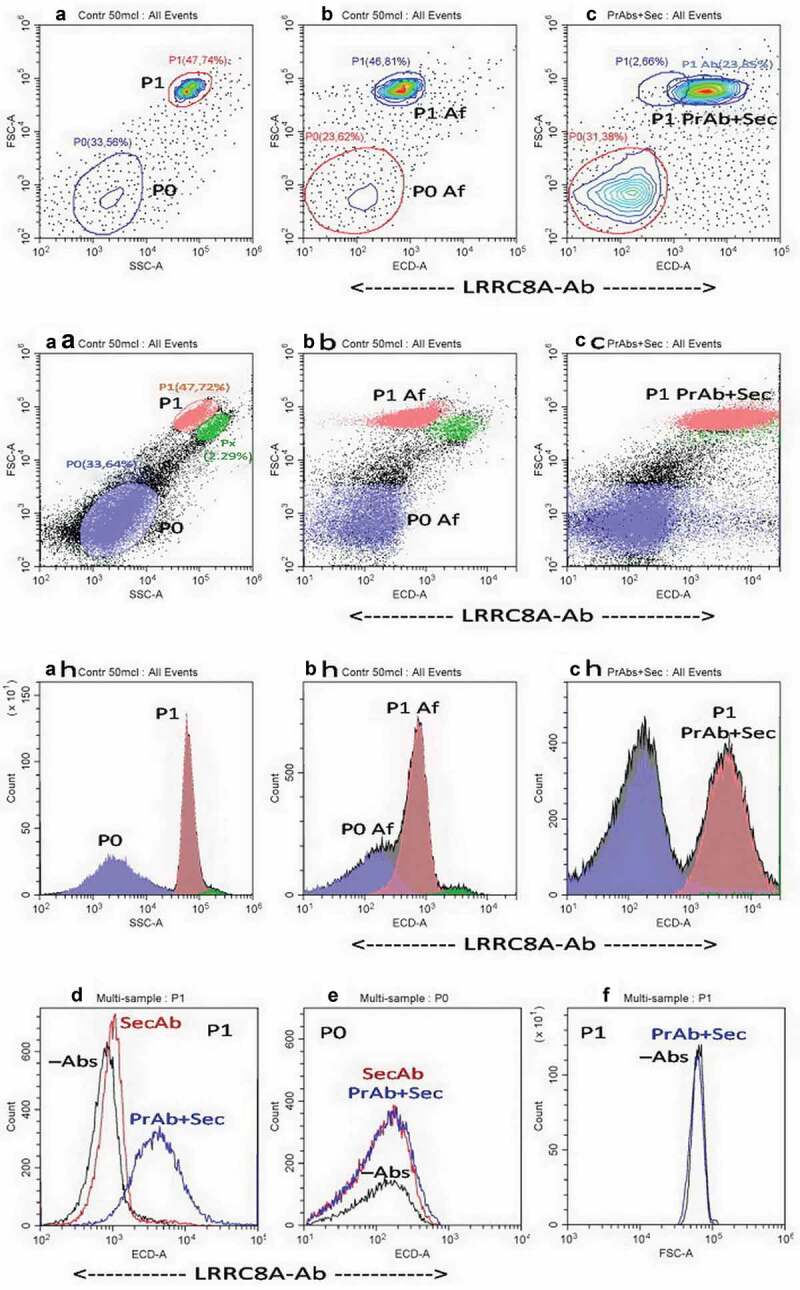
(a)Differentiation of whole cells (subset P1) and microparticles (subset P0) in unstained U937 cultures by analysis of FSC/SSC contour diagrams. (b) FSC/ECD diagram of unstained cells revealing background autofluorescence (Af) of P1 and P0 particles in ECD channel specific for the LRRC8A-Ab, (c) Contour diagram for sample stained with primary+secondary Ab (PrAb+Sec). (aa, bb, cc) Dot plots and (ah, bh, ch) histograms of the data presented above as contour plots, since many readers prefer dot plots. A small subset Px is visible on the dot plots, the share of which is only 2.3 % and is not discussed here. (d, e) P1 and P0 subset histograms for unstained (–Abs), stained with only secondary (SecAb) and with primary+secondary Ab (PrAb+Sec). Each pair of P1 and P0 plots in Figs d, e corresponds to the same sample. (f) FSC histograms of P1 subset obtained for the same samples as histograms for LRRC8A-Ab specific channel ECD. X-axes in d-f panels have the same scale extension. Different treatments correspond to different samples but are experimentally controlled for time and cultured cell line. Representative data of one of three independent experiments are shown.

In addition to whole cells, flow cytometry detects many cellular particles including cell fragments, microparticles, and cell derivatives like apoptotic bodies. First, we explored how the quantity of the bound Ab depends on the size of cells, cell fragments and other microparticles. Forward and side light scattering (FSC, SSC) are established surrogate indicators of particle size in flow cytometry [20,21]. Figure 1(a) shows the routine FSC/SSC contour diagrams of an original U937 cell culture, revealing two main subsets of particles designated as P0 and P1. The P0 subset contains a central area surrounded by chaotically distributed microparticles. Earlier we’ve shown that apoptosis increases the P0 subset suggesting these particles contain apoptotic bodies. Thus, even in unmanipulated U937 cells, microparticles formed by cells during apoptosis may be spontaneous and unavoidable [22]. The P1 subset contains mostly healthy, whole cells as confirmed by our previous work via annexin and propidium iodide tests [22]. Subsets P0 and P1 are clearly seen in FSC/ECD contour plots (Figure 1(b)) where ECD channel is used for specific visualizing the LRRC8A-Ab. This allows us to control for the background autofluorescence just in the proper channel. Diagrams in Figure 1(b,bb) indicate that autofluorescence diagrams are about the same as FSC diagrams both for P0 particles and P1 cells. This is also the case for SSC signals.

### Binding of LRRC8A Ab to whole cells (P1) is visible and significant

Figure 1(c,cc,d) demonstrate that fluorescence of the whole cells (subset P1) with bound LRRC8A-Ab is much higher than the background autofluorescence of unstained cells or the cells contacted only with the secondary Ab. Fluorescence of the P0 particles from the same sample did not differ notably from the background autofluorescence (Figure 1(e)). This could be due to the small size of P0 particles as well as their relatively high autofluorescence. The latter is revealed by comparing P0 and P1 autofluorescence signals “normalized” with size marker signal, e.g. SSC or FSC signal. The mean Af/FSC ratio obtained by CytExpert software for P1 and P0 is 0.1 and 2.1, respectively, where Af is the autofluorescence in LRRC8A specific ECD channel.

**Figure 2.**
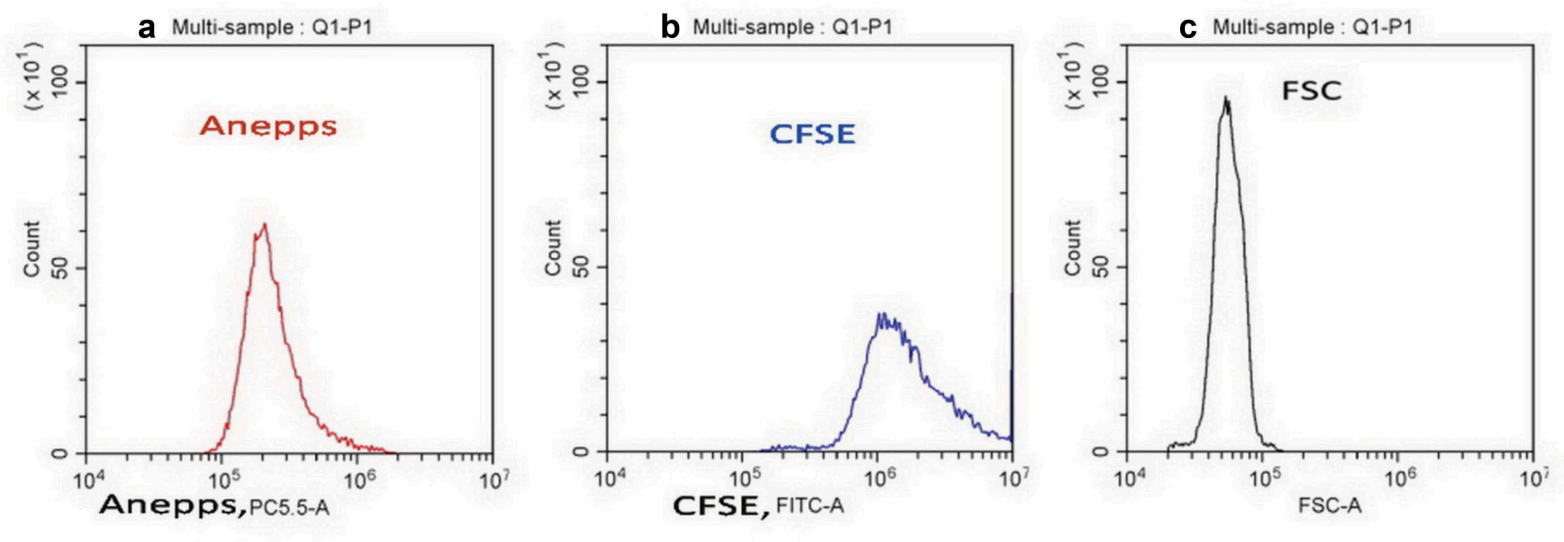
The distribution of cells (P1) of the same sample stained with both ANEPPS (a) and CFSE (b), determined by their fluorescence in channels PC5.5 (ANEPPS) and FITC (CFSE), and the corresponding FSC histogram (c). Representative data of one of three independent experiments are shown.

No direct correlation was found between quantity of LRRC8A-Ab bound to whole cells and the cell size. The distribution of cells labeled with LRRC8A-Ab differs markedly from that of the cell size indicators, FSC and SSC (Figure 1). The FSC/ECD coordinates for the P1 cluster of cells stained with the LRRC8A-Ab (Figure 1) indicates that cell dispersion by bound antibody quantity is wider than dispersion by FSC. Another indication of this difference is the comparison of the respective histograms (Figure 1(d,f)). Further, the P1 LRRC8A-Ab cluster dispersion is wider also than that for autofluorescence (Figure 1(d)). Probes of other types were investigated in comparison with the LRRC8A-Ab. The cell distribution by quantity of lipophile dye ANEPPS, which may be a “surface” marker, or dye CFSE, considered often as a “cytoplasm” marker, are also not as broad as the LRRC8A-Ab cluster (Figure 2). Thus, we suggest that quantity of cell surface LRRC8A-VRAC channels in the studied population of U937 cells is determined by factors other than the size of the area or volume of a sphere imitating a cell.

### U937 cell exposure to STS or hypotonic medium alter cell size but not surface VRAC expression

**Figure 3.**
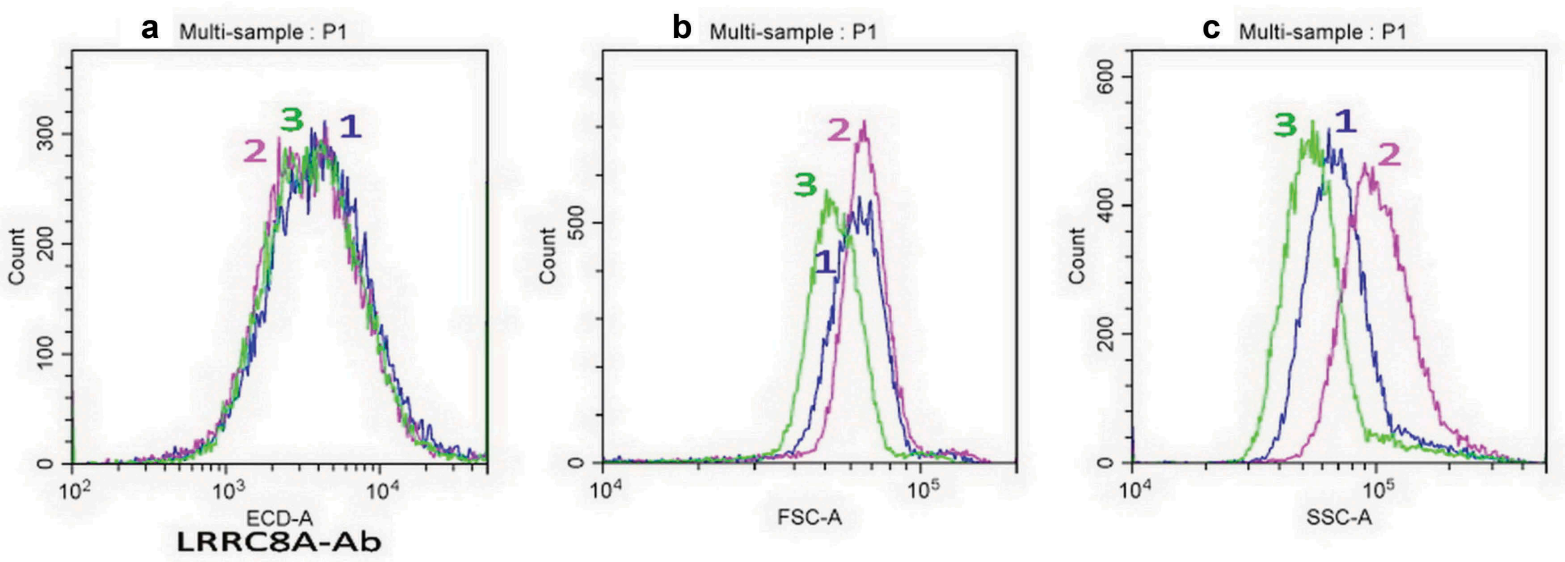
No effect of STS or hypotonic medium on anti-LRRC8A Ab histograms (a) in cells responding by changes in FSC (b) and SSC (c). P1 subset histograms of cells, stained with LRRC8A-Ab+secondary Ab (1) and additionally treated for 1 h with 1 µM STS (2) or with hypotonic medium (3). Each of 1–3 histograms corresponds to separate sample. The compared histograms were obtained in the same experiments. Representative data of three independent experiments are shown.

Recently, we showed that STS-apoptosis of U937 cells induced a 5-fold increase in the integral permeability of chloride channels during the first hour [3]. Furthermore, many electrophysiological data obtained in various cells indicate that LRRC8A-VRAC respond to hypotonic media by an increasing chloride current. For that reason, we compared changes in membrane-bound LRRC8A-VRAC in U937 cells following STS-induced apoptosis or hypotonicity. No difference in the quantity of bound LRRC8A-Ab in U937 cells induced to apoptosis or treated with hypotonic media for 1 h and untreated control cells was revealed (Figure 3). The similarity in the LRRC8A-Ab histograms for all three conditions indicates that the dosage accuracy of the antibodies added to the different samples and the reproducibility of the measurements were satisfactory. Therefore, it would be possible to detect differences if indeed they had been consistent with changes in the integral chloride channels permeability stated before in apoptotic U937 cells under similar conditions by ion assay [3]. Remarkably, the effects of STS-induced apoptosis and hypotonic medium treatment of cells were clearly seen in FSC and SSC histograms (Figure 3(b,c)) of the same samples. These FSC and SSC data are consistent with our previous findings wherein STS and modified water content alter cell size [22].

## Discussion

VRAC operates as a heteromeric complex of several proteins from the LRRC8 family. Since the molecular structure of VRAC was identified, a series of antibodies against different regions of the basic subunits of the family LRRC8 have been developed and effectively used [4,5,9,14,17,18,23–26]. Before now, VRAC subunit proteins were detected mostly by immunoblotting of the solubilized whole cells [27–32], less often of cell membranes lysates [14,33,34] or by imaging cells that have been permeabilized for staining [35,36]. Furthermore, these data were mostly obtained from cells specifically rich in VRAC such as epithelial cells, astrocytes, vascular smooth muscle cells and transfected cells.

Surface localization of LRRC8A has been shown in several cases [4,36–38]. Intracellular and plasma membrane localization of GFP-tagged LRRC8A was visualized in living KCP-4 and KB cell lines by immunoimaging [30]. Geha and colleagues used flow cytometry to show specific binding of antibodies against the extracellular loop (87–104 amino acids) of LRRC8A in mice splenocytes. However, the researchers stained the cells after permeabilization, thus the results included intracellular LRRC8A expression [31].

The new commercially available antibody, AAC-001, was developed by the Alomone Lab to be a promising tool for studying surface expression of LRRC8A in living cells using flow cytometry. These antibodies are generated against the first extracellular loop (91–104 amino acids) of LRRC8A, which is mandatory for a functional VRAC complex [18,39]. To our knowledge, the surface expression of endogenous LRRC8A in living cells has not been explored until now.

For many years, the U937 cell line has been a standard for studying cellular functions. We have used U937 cells for studying the role of monovalent ions in apoptosis [3,22,40–43]. Recently, our measurements and calculations of the monovalent ion flux balance [3] have led us to conclude that shrinkage of cells specifically during the initial stage of apoptosis (apoptotic volume decrease, AVD) is associated with the complex alteration of channels and transporters carrying monovalent ions across the plasma membrane. STS-induced apoptosis of U937 cells leads to a decrease in the Na^+^/K^+^-ATPase rate coefficient, as well as changes in sodium and potassium channels permeability, and, most importantly for the present study, increases the integral permeability of the chloride channels during the first hour by approximately 5-fold.

We explored the role of surface VRAC expression following cell stressors known to cause increased chloride permeability by quantifying bound LRRC8A-Ab on living whole cells via flow cytometry. We were able to measure surface expression of the endogenous LRRC8A-VRAC in living cells that are not specifically rich in these channels without using over-expression of the tagged channels by transfection. We revealed that the number of LRRC8A subunits did not changed on U937 cells under the conditions studied despite the robust changes in chloride channel permeability reported in our previous study [3]. Alomone Lab checks specificity of each antibody lot by Western blot analysis and there is no doubt in their quality. It’s more likely that the number of VRAC channels doesn’t really change, and the chloride flow through the channels is modulated in another way. Translocation of the chloride ion current inducer protein, ICln, from the cytosol into the cell membrane for 10–20 minutes after initial hypotonic shock has been shown for various cells [13,44].

Our findings are more consistent with those of Lambert and colleagues who have explored long-term regulation of VRAC-LRRC8 channels at the transcriptional, translational and functional level [33,34]. This group has described many situations where long-term changes in functional channel activity were caused by modifications of the LRRC8A protein but not by changes in its quantity in whole cell or the plasma membrane. Their general consensus is that “modulation of osmolyte transport via the VRAC complex is mainly controlled through (1) posttranslational modulation of LRRC8 subunits, (2) shift in the stoichiometry between LRRC8A and other LRRC8 family members, (3) modulation of the number of functional VRAC complexes in the plasma membrane, and (4) shift in the activity of signaling cascades involved in modulation of VRAC complex activity” [33]. Thus, it is likely that following STS or osmotic stress, many complex variables interact to alter chloride flux. Importantly, it may be possible to examine these possibilities in living, whole cells with improved development of antibody-flow cytometry systems.
